# Acute Exercise-Induced Mitochondrial Stress Triggers an Inflammatory Response in the Myocardium via NLRP3 Inflammasome Activation with Mitophagy

**DOI:** 10.1155/2016/1987149

**Published:** 2015-12-06

**Authors:** Haiying Li, Weiguo Miao, Jingfen Ma, Zhen Xv, Hai Bo, Jianyu Li, Yong Zhang, Li Li Ji

**Affiliations:** ^1^Tianjin Institute of Health and Environmental Medicine, Tianjin 300050, China; ^2^Tianjin Key Laboratory of Exercise Physiology and Sports Medicine and Department of Health and Exercise Science, Tianjin University of Sport, Tianjin 300381, China; ^3^Logistics College of People's Armed Police Forces, Tianjin 300162, China; ^4^Laboratory of Physiological Hygiene and Exercise Science, School of Kinesiology, University of Minnesota, Minneapolis, MN 55455, USA

## Abstract

Increasing evidence has indicated that acute strenuous exercise can induce a range of adverse reactions including oxidative stress and tissue inflammation. However, little is currently known regarding the mechanisms that underlie the regulation of the inflammatory response in the myocardium during acute heavy exercise. This study evaluated the mitochondrial function, NLRP3 inflammasome activation, and mitochondrial autophagy-related proteins to investigate the regulation and mechanism of mitochondrial stress regarding the inflammatory response of the rat myocardium during acute heavy exercise. The results indicated that the mitochondrial function of the myocardium was adaptively regulated to meet the challenge of stress during acute exercise. The exercise-induced mitochondrial stress also enhanced ROS generation and triggered an inflammatory reaction via the NLRP3 inflammasome activation. Moreover, the mitochondrial autophagy-related proteins including Beclin1, LC3, and Bnip3 were all significantly upregulated during acute exercise, which suggests that mitophagy was stimulated in response to the oxidative stress and inflammatory response in the myocardium. Taken together, our data suggest that, during acute exercise, mitochondrial stress triggers the rat myocardial inflammatory response via NLRP3 inflammasome activation and activates mitophagy to minimize myocardial injury.

## 1. Introduction

Growing evidence suggests that the exercises of varying intensities have distinct effects on the body. Numerous reports have revealed that moderate physical activity enhances the function of various organs and tissues [1]. In contrast, an acute bout of heavy exercise can induce a range of adverse effects including inflammatory response and oxidative stress. Many investigators found that inflammatory cytokines were involved in activating catabolic pathways during an acute bout of exhausting exercise [2]. Although the majority of studies have been conducted in the skeletal muscle, there is evidence that heart can also be subjected to oxidative stress during strenuous physical work under the influence of blood-borne stress hormones (adrenalin and glucocorticoids), proinflammatory cytokines, and locally produced prooxidants [3]. However, the detailed regulatory mechanism involved in the inflammatory response of the myocardium during acute exercise remains unclear.

Mitochondria, the primary energy-producing organelles, play a central role in cellular survival and death. However, mitochondria have recently been found to function as an important source of damage-associated molecular patterns (DAMPs) such as mtDNA, cytochrome C, and reactive oxygen species (ROS) in inflammatory responses of the myocardium [4, 5]. It has been demonstrated that dysfunctional mitochondria can release DAMPs and enhance inflammatory response, for which activation of the NLRP3 inflammasome is required [6]. The NLRP3 inflammasome, a large multiprotein complex, plays a key regulatory role in the pathogenic processes of the inflammation by modulating its downstream target, IL-1*β* [7]. The NLRP3 inflammasome is activated by a variety of danger signals integrated from the mitochondria, including infection, metabolic dysfunction, and oxidative stress [8]. Thus, we speculate that mitochondrial stress would trigger NLRP3-induced inflammatory response in the myocardium during acute exercise.

Autophagy is a “self-eating” process that degrades cellular components. Similarly, mitophagy is a catabolic process involved in the removal of dysfunctional mitochondria and a key player in the negative regulation of the NLRP3 inflammasome [9]. Previous evidence has shown that mitophagy is an essential process for maintaining normal cardiac function during stress via the removal of damaged or misfolded mitochondrial proteins [10]. It also serves as a protective response activated by drugs against cardiac injury. For example, simvastatin has been proven to trigger Parkin-dependent mitophagy thereby conferring cardioprotection [11]. Thus, we speculate that an acute bout of exercise may activate mitophagy as a protective response to cardiac inflammatory reactions.

Overall, the current study had the following aims: (1) to investigate the effects of mitochondrial stress on the inflammatory responses of the myocardium during various durations of acute exercise and the subsequent recovery period and (2) to examine the role of NLRP3 inflammasome in the inflammatory responses and the regulation of mitophagy to acute exercise in the myocardium.

## 2. Methods

### 2.1. Animals and Groups

Male Sprague-Dawley rats (age 8 weeks) were housed in a temperature-controlled (21-22°C) room. The Institutional Review Board of the Tianjin University of Sport approved all experiments under the guidelines of the Chinese Academy of Sciences. The rats were randomly divided into eight groups (*n* = 8 for each group): the resting control group (RC), the 45 min exercise group (E-45), the 90 min exercise group (E-90), the 120 min exercise group (E-120), the 12 h postexercise recovery group (PE-12), the 24 h postexercise recovery group (PE-24), the 36 h postexercise recovery group (PE-36), and the 48 h postexercise recovery group (PE-48).

### 2.2. Acute Exercise Model

According to Bedford et al.'s study [12], the rats were subjected to an acute bout of incremental treadmill running for various durations and the maximum oxygen consumption (VO_2_max) of rats was calculated on a body mass basis. All groups started running on a motor-driven treadmill for 15 min at 8.2 m/min, 0° grade (~53% VO_2_max), followed by 15 min at 15 m/min, 5° grade (~64% VO_2_max). Thereafter, three groups of rats ran at 19.3 m/min, 10° grade (~76% VO_2_max) continuously for 15, 60, or 90 min and were sacrificed immediately by injection of pentobarbital sodium. Four additional groups of rats performed the exercise protocols described above and were killed at 12, 24, 36, or 48 h after running for a total of 120 min.

### 2.3. HE Staining

The left ventricles of rats were isolated and the paraffin-embedded sections were prepared, dewaxed, and then incubated in the solution of hematoxylin for 5 min, followed by differentiation with acid alcohol. The nuclei were subsequently stained with ammonia water. Next, the slides were incubated in the eosin solution for 2 min. After dehydration, clearing, and mounting, the sections were observed using a light microscope (Nikon E200, Tokyo, Japan).

### 2.4. Heart Mitochondrial Isolation

The left ventricular mitochondria of rats were prepared using differential centrifugation according to established protocols [13]. Briefly, the minced blood-free tissue was homogenized in the buffer solution containing 0.25 M sucrose, 3.0 mM Hepes, and 0.5 mM EDTA (pH 7.4). The homogenate was centrifuged at 800 ×g for 10 min, and the resulting supernatant was centrifuged at 10,000 ×g for 10 min. The pellets were gently resuspended and centrifuged again at 10,000 ×g for 10 min, and the final pellets were suspended in 1 mL of the isolation medium. The mitochondrial protein content was detected using the Bradford assay.

### 2.5. Mitochondrial Respiration

Mitochondrial respiratory function was measured using a high-resolution respirometer, Oxygraph-2k (Oroboros Co., Austria). The reactions were conducted at 37°C in a thermostated and magnetically stirred chamber containing 130 mM KCl, 10.0 mM Hepes, 1 mM EDTA, 2.5 mM KH_2_PO_4_, and 1 mg/mL BSA (pH 7.4) with 0.3 mg mitochondrial protein. After a 10 min equilibration period, mitochondrial respiration was initiated by adding 2 M pyruvic acid sodium and 0.8 M malate. After a stable state 2 respiration (ST2) was established, a state 3 respiration (ST3) was initiated by the addition of 0.5 M ADP. After all of the added ADP had been phosphorylated to ATP, the respiratory rate returned to state 4. The respiratory control ratio (RCR) was calculated as the ratio of the respiratory rate in state 3 to that in state 4.

### 2.6. Mitochondrial Membrane Potential and ATP Synthase Activity

The isolated mitochondrial membrane potential (Δ*ψ*) was measured by monitoring the fluorescence spectrum of JC-1 [14]. The experiments were performed at 37°C in an incubation medium containing 1.5 mL JC-1 staining solution, 2 M pyruvic acid sodium, and 0.8 M malate with 0.3 mg mitochondrial protein. ATP synthase activity was determined using a bioluminescence technique [15]. The mitochondrial suspensions were added to a cuvette containing 40 *μ*M luciferase (Sigma, MO, USA), 0.25 M sucrose, 3.0 mM Hepes, 0.5 mM EDTA, 2 M pyruvate, and 0.8 M malate. After a background bioluminescence was established for correction, 0.5 *μ*M ADP was added to initiate the reaction. ATP production was monitored at 37°C using a BioOrbit 20/20n luminometer (Turku, Finland) and expressed as nanomoles per minute per mg protein.

### 2.7. Mitochondrial ROS Production

The mitochondrial ROS generation rate was monitored using a dichlorofluorescein diacetate (H2-DCFDA) probe [16]. Briefly, the H2-DCFDA stock solution was dissolved in 1.25 mM methanol in a dark room at 0°C. To initiate the experiment, 0.3 mg mitochondrial protein was added to a quartz cuvette containing 2.5 mL of 0.1 M phosphate buffer (pH 7.4) and 2 *μ*L of 2.5 mM H2-DCFDA. The assay mixture was incubated at 37°C for 15 min. DCF formation was determined fluorometrically at an excitation wavelength of 499 nm and an emission wavelength of 521 nm at 37°C for 2 min, using a Cary Eclipse fluorescence spectrophotometer (Varian, CA, USA). The units were expressed as a picomole of DCF formed per minute per mg of protein.

### 2.8. Mitochondrial MnSOD Activity and MDA Content

SOD activities were measured using the method [17], where one unit of SOD activity was defined as the amount of enzyme that causes a 50% inhibition of epinephrine autoxidation to adrenochrome. To determine the MnSOD activity, 1 mM KCN^−^ was added to the reaction mixture to inhibit the activity of CuZnSOD. The content of MDA was measured using the thiobarbituric acid method. In brief, the MDA in mitochondrial samples reacted with thiobarbituric acid and a pink-colored product produced in the reaction. For detecting the contents of MDA, the reaction products were quantified as the absorbance at 532 nm according to the manufacturer's instructions.

### 2.9. Western Blotting Analysis

The left ventricular tissue samples of rats were freshly homogenized in the lysis buffer containing protease inhibitor PMSF (phenylmethanesulfonyl fluoride). The homogenates were solubilized using end-over-end mixing for 60 min at 4°C followed by centrifugation at 15,000 ×g for 20 min at 4°C. The total protein concentration was determined using the Bradford assay. The whole-cell proteins solubilized in the sample buffer were separated using SDS-PAGE and transferred to PVDF membranes (Millipore, MA, USA). The membranes were blocked and exposed to primary rabbit polyclonal antibodies (anti-Bnip3, 1 : 500; anti-Beclin1, 1 : 1000; anti-LC3, 1 : 500; anti-IL-1*β*, 1 : 1000; and anti-*β*-tubulin, 1 : 3000) (Abcam, Cambridge, UK) and primary goat polyclonal antibody (anti-NLRP3, 1 : 500) (Abcam, Cambridge, UK) overnight at 4°C. The membranes were washed and incubated with the HRP-conjugated anti-rabbit IgG (1 : 10000) and the HRP-conjugated anti-goat IgG (1 : 10000) (CST, Boston, USA) for 60 minutes. The data were analyzed using Quantity One software (Bio-Rad, CA, USA) to obtain the optical density ratio of target proteins relative to *β*-tubulin.

### 2.10. Statistical Analyses

The data were presented as the mean ± SEM and analyzed using a one-way ANOVA followed by Dunnett's many-to-one test. Statistical Package for the Social Sciences (SPSS Inc., version 15.0) was used for all analyses. The significance level was set at *p* < 0.05.

## 3. Results

### 3.1. Pathological Assessment

**Figure 1:**
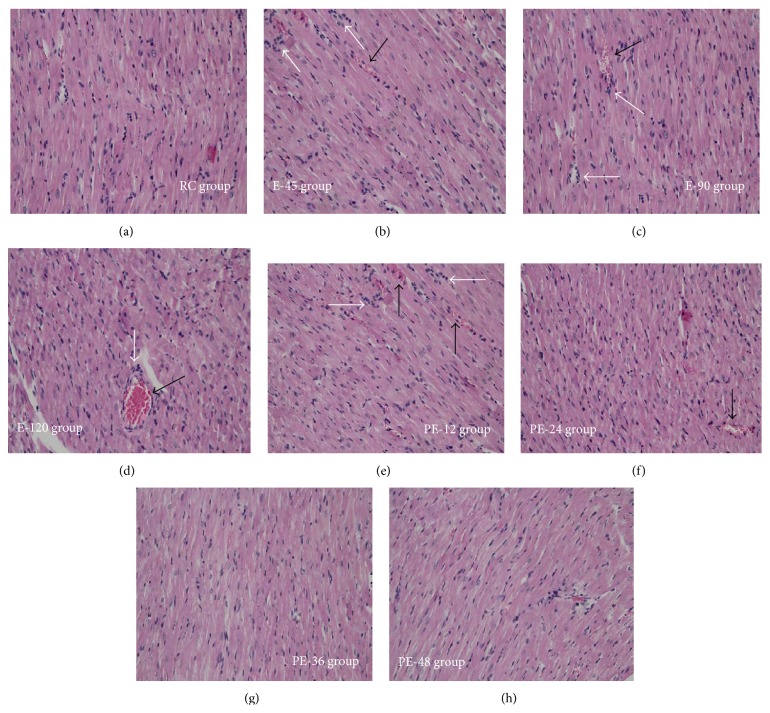
The pathological changes of the rat myocardium during acute exercise were observed using HE staining. Compared with those of the RC group (a), more dilatation and congestion of capillaries (the highlight of the black arrows) and inflammatory cell infiltration (the highlight of the white arrows) were observed in the cardiac tissues during the exercise period ((b), (c), and (d)) and the early stage of recovery ((e) and (f)).

In this study, no apparent changes in the morphology or structure of the myocardial tissues were found during acute exercise and during the recovery; however, myocardial tissues were found to display more dilatation and congestion of capillaries and inflammatory cell infiltration in the E-45, E-90, and E-120 groups, compared with those of the RC group. Moreover, characteristic inflammatory responses in the rat myocardium were also observed during the early stage of recovery in the PE-12 and PE-24 groups (Figure 1).

### 3.2. Mitochondrial Respiratory Function in the Myocardium

**Figure 2:**
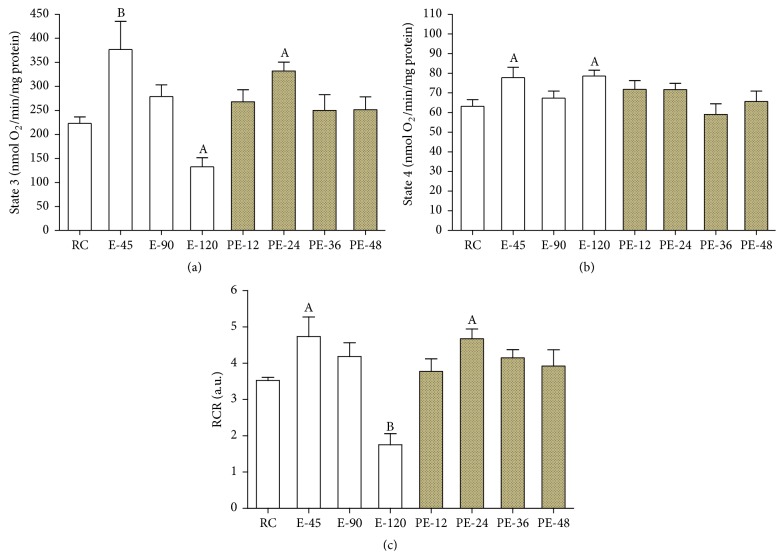
To investigate the mitochondrial respiratory function in the myocardium, mitochondrial ST3, ST4, and RCR were determined. Compared with that of the RC group, mitochondrial ST3 and RCR were both increased during the early stages of both acute exercise and its recovery ((a) and (c)), and ST4 was increased only during acute exercise (b). Each bar represents the mean ± SEM of a treatment group (*n* = 8). ^A^ *p* < 0.05 and ^B^ *p* < 0.01 versus RC group.

Compared with that of the RC group, mitochondrial ST3 respiration rate was elevated by 68.9% (*p* < 0.01) in the E-45 group but decreased by 40.6% (*p* < 0.05) in the E-120 group. During the recovery, ST3 increased by 48.9% again (*p* < 0.05) in the PE-24 group before returning to the RC level in the PE-36 and PE-48 groups (Figure 2(a)). ST4 respiration rate was increased by 23.1% and 24.3% (*p* < 0.05) in both E-45 and E-120 groups and then returned to the RC level in all PE groups (Figure 2(b)). Similar to ST3 respiration rate, RCR was increased by 34.3% in the PE-45 group (*p* < 0.05), decreased by 50.3% in the E-120 group (*p* < 0.01), and rebounded by 32.6% in the PE-24 group (*p* < 0.05) (Figure 2(c)).

### 3.3. Mitochondrial Membrane Potential and ATP Synthase Activity

**Figure 3:**
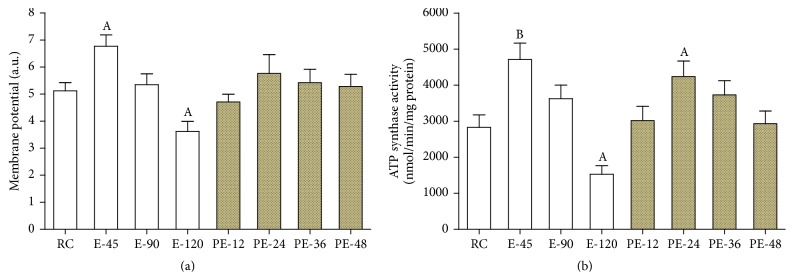
To investigate the mitochondrial stress response in the myocardium, mitochondrial membrane potential and ATP synthase activity were determined. Compared with that of the RC group, mitochondrial membrane potential and ATP synthase activity were both increased during the early stages of acute exercise and/or its recovery ((a) and (b)). Each bar represents the mean ± SEM of a treatment group (*n* = 8). ^A^ *p* < 0.05 and ^B^ *p* < 0.01 versus RC group.

Compared with that of the RC group, mitochondrial membrane potential was increased by 32.2% (*p* < 0.05) in the E-45 group but decreased by 29.4% (*p* < 0.05) in the E-120 group; it returned to the RC level in all PE groups (Figure 3(a)). Mitochondrial ATP synthase activity was elevated by 66.6% (*p* < 0.01) in the E-45 group but decreased by 45.9% (*p* < 0.05) in the E-120 group. During the recovery, ATP synthase activity increased by 50.0% again (*p* < 0.05) in the PE-24 group before returning to the RC level in the PE-36 and PE-48 groups (Figure 3(b)).

### 3.4. Mitochondrial Prooxidants and Antioxidants

**Figure 4:**
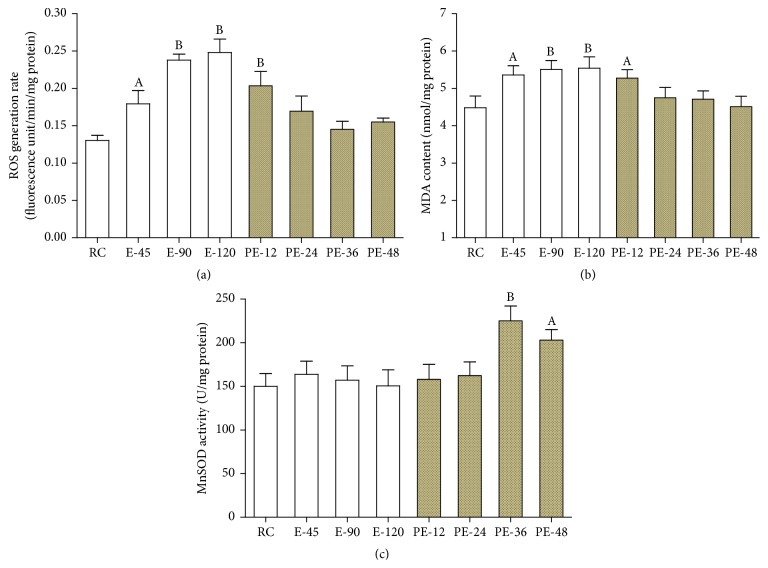
To investigate the mitochondrial oxidative stress response in the myocardium, ROS generation rate, MnSOD activity, and MDA content were measured. Compared with that of the RC group, mitochondrial ROS generation and MDA content were both increased during acute exercise and early recovery ((a) and (b)), but MnSOD activity was not increased until late recovery (c). Each bar represents the mean ± SEM of a treatment group (*n* = 8). ^A^ *p* < 0.05 and ^B^ *p* < 0.01 versus RC group.

Compared with that of the RC group, the mitochondrial ROS generation rate was increased by 37.5%, 82.6%, and 90.4% in the E-45 (*p* < 0.05), E-90 (*p* < 0.01), and E-120 (*p* < 0.01) groups during acute exercise and also enhanced by 56.0% (*p* < 0.01) during the early stage of recovery (PE-12 group). It then returned to the RC level in the PE-24, PE-36, and PE-48 groups (Figure 4(a)). Mitochondrial MDA content was increased by 19.5%, 22.8%, and 23.6% in the E-45 (*p* < 0.05), E-90 (*p* < 0.01), and E-120 (*p* < 0.01) groups during acute exercise and also enhanced by 17.7% (*p* < 0.05) during the early stage of recovery (PE-12 group). It then returned to the RC level in the PE-24, PE-36, and PE-48 groups (Figure 4(b)). MnSOD activity did not change during the exercise period or the early stage of recovery (PE-12 and PE-24 groups) and then was increased by 50.1% and 35.3% in the PE-36 (*p* < 0.01) and PE-48 (*p* < 0.05) groups (Figure 4(c)).

### 3.5. The Expressions of NLRP3 and Its Downstream Target IL-1*β*

**Figure 5:**
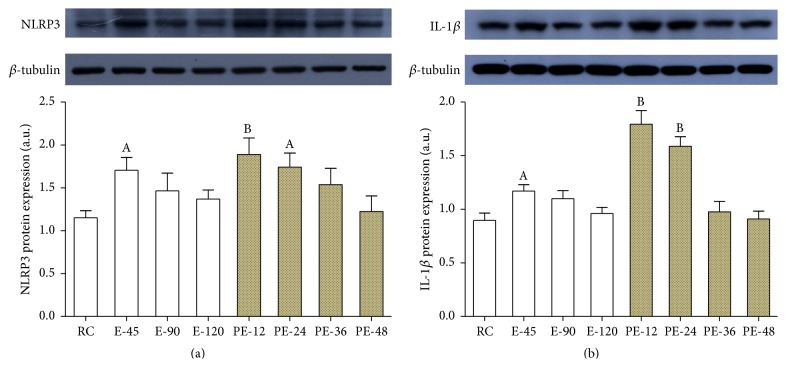
To investigate the inflammatory response in the myocardium, the expressions of NLRP3 and its target IL-1*β* were determined. The data showed that the expressions of NLRP3 and IL-1*β* were upregulated in the early stage of both acute exercise and its recovery ((a) and (b)). Each bar represents the mean ± SEM of a treatment group (*n* = 8). ^A^ *p* < 0.05 and ^B^ *p* < 0.01 versus RC group.

NLRP3 protein content was increased by 48.1% in E-45 versus RC (*p* < 0.05), but the change was not significant in E-90 or E-120. During the recovery, NLRP3 level was increased by 63.9% and 51.2% again in PE-12 (*p* < 0.01) and PE-24 (*p* < 0.05) before returning to RC level (Figure 5(a)). IL-1*β* expression was upregulated by 30.5% in E-45 versus RC (*p* < 0.05), but the change was not significant in E-90 or E-120. During the recovery, IL-1*β* level was enhanced by 100.0% and 77.0% again in PE-12 and PE-24 (*p* < 0.01) before returning to RC level (Figure 5(b)).

### 3.6. The Expressions of Mitochondrial Autophagy-Related Proteins

**Figure 6:**
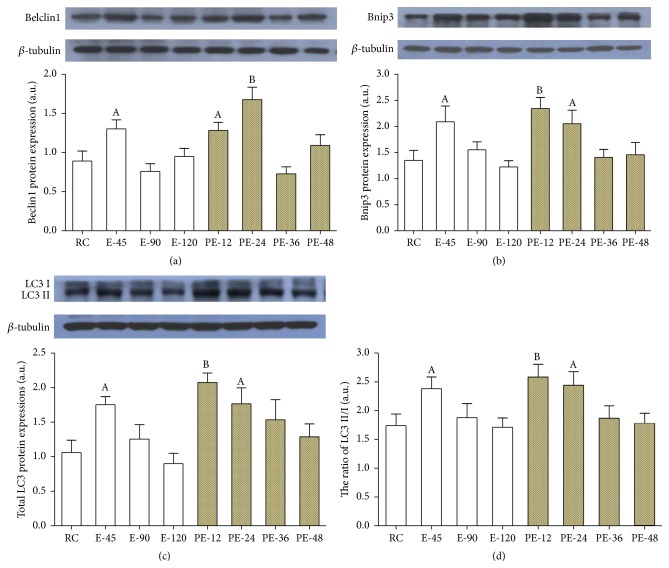
To investigate the mitophagy in the myocardium, the expressions of several mitochondrial autophagy-related proteins were examined. Compared with that of the RC group, the expressions of Beclin1, Bnip3, and total LC3, as well as the ratio of LC3 II to LC3 I, were all increased during the early stages of both acute exercise and its recovery ((a), (b), (c), and (d)). Each bar represents the mean ± SEM of a treatment group (*n* = 8). ^A^ *p* < 0.05 and ^B^ *p* < 0.01 versus RC group.

Beclin1 expressions were enhanced by 46.2% in E-45 versus RC (*p* < 0.05), but they returned to the RC level in the E-90 and E-120 groups. During the recovery, Beclin1 level was increased by 43.9% and 88.2% again in the PE-12 (*p* < 0.05) and PE-24 (*p* < 0.01) groups before returning to the RC level (Figure 6(a)). Bnip3 protein content was upregulated by 55.1% in E-45 versus RC (*p* < 0.05), but the change was not significant in E-90 or E-120. During the recovery, Bnip3 level was increased by 73.9% and 52.4% again in the PE-12 (*p* < 0.01) and PE-24 (*p* < 0.05) groups before returning to the RC level (Figure 6(b)). The expressions of total LC3 increased by 65.2% and the ratio of LC3 II to LC3 I increased by 36.7% in the E-45 group (*p* < 0.05) but returned to the RC level in the E-90 and E-120 groups. During the recovery, the expressions of total LC3 increased by 95.5% and 66.5% again in the PE-12 (*p* < 0.01) and PE-24 (*p* < 0.05) groups, and the ratio of LC3 II to LC3 I increased by 48.3% and 40.1% again in the PE-12 (*p* < 0.01) and PE-24 (*p* < 0.05) groups before returning to the RC level (Figures 6(c) and 6(d)).

## 4. Discussion

Accumulating evidence has shown that moderate exercise can result in an increased resistance to oxidative challenge by maintaining the balance between prooxidants and antioxidants [18]. On the other hand, strenuous exercise (e.g., acute fatiguing exercise) can promote the excessive generation of ROS and lead to transient immunosuppressant and inflammatory responses [19].

Oxidative stress and inflammation have been found to be associated with an excess of ROS, which could potentially be a main source of mitochondrial dysfunction. Recent studies have found that the damage of muscle tissue arising from strenuous exercise increases the level of ROS and induces an inflammatory response [20]. Regarding the sources of ROS in exercise, it is generally accepted that the superoxide is mainly produced by mitochondria, phagocytes, and xanthine oxidase (XO) [21]. During exercise, one of the most common forms of ROS generation is due to electron leaks from the mitochondrial electron transport chain (ETC). In this research, we used the DCFH-DA probe to measure the generation of mitochondrial ROS and used the TBARS assay to detect MDA content in mitochondria during acute exercise. We acknowledge the limitations in the above methods as have been reported [22, 23]; however, the DCFH-DA probe and the TBARS assay continue to be used for the assessment of ROS and MDA level [24, 25]. The results of the current study showed that the ROS generation and MDA content in the mitochondria of rat myocardium significantly increased during acute exercise and early recovery; however, the antioxidant enzyme MnSOD in mitochondria was not enhanced until 36 and 48 hours after exercise. NLRP3, an important inflammatory factor, is implicated in various inflammatory responses, and its function primarily depends on a multiprotein complex called the inflammasome. The formation of the NLRP3 inflammasome and the production of its downstream target IL-1*β* are generally thought to be responsible for the mitochondrial ROS-mediated inflammatory reaction to a variety of stresses [26]. It has been demonstrated that ROS production derived from mitochondria in the myocardium could induce the oligomerization of NLRP3, recruitment of apoptosis-associated speck-like protein containing a CARD domain (ASC), and activation of caspase-1 [27]. The activation of caspase-1 can transform pro-IL-1*β* to mature IL-1*β*, leading to enhanced inflammation and mitochondrial dysfunction [28]. Finally, damaged mitochondria may produce greater amount of ROS and form a vicious cycle of inflammatory response [29]. This scenario is consistent with our data showing that the upregulated expressions of NLRP3 and IL-1*β* took place at the early stage of exercise (E-45), followed by a surge of ROS production. Furthermore, morphological parameters such as the dilatation and congestion of capillaries as well as inflammatory cell infiltration were observed in the rat myocardium, providing further support for the above point of view. These results suggest that NLRP3 inflammasome activation and subsequent inflammatory response might play a key role in explaining the mitochondrial stress response in the myocardium, such as enhanced ST3 respiration and RCR, as well as ATP production in rats exercised for two hours (Figures 2(a), 2(c), and 3(b)).

To investigate the detailed mechanism involved in the regulation of the inflammatory response of the myocardium during acute exercise, mitochondrial functions of the rat myocardium, including the ATP synthase activity, membrane potential, and respiratory function of mitochondria, were determined. ATP generation should be required to supply energy to maintain the dynamics of myocardial contraction during acute heavy exercise. In this study, our data indicated that the activities of mitochondrial ATP synthase were enhanced during the early stages of both acute exercise and its recovery, and this finding was accompanied by increased levels of ROS and the upregulation of inflammatory factors. These effects suggest that the enhanced process of oxidative phosphorylation promoted the consumption of oxygen, induced the overproduction of ROS, and stimulated the mitochondrial ROS-mediated inflammatory response.

In addition to increased ATP production, we found that the membrane potential of mitochondria was elevated during the mitochondrial stress response associated with the early stage of acute exercise. Increased mitochondrial membrane potential can promote the formation of ROS, and the levels of ROS were exponentially enhanced at membrane potentials above 140 mV [30]. Thus, we assumed that the elevated membrane potential of mitochondria might precipitate increased ROS generation and the inflammatory response during acute exercise. Mitochondrial RCR, calculated as ST3 divided by ST4, is a critical parameter of mitochondrial respiratory function and decreases in this parameter are involved in the dysfunction of mitochondria in aging and various diseases [31]. However, the data in the current study revealed that mitochondrial RCR was enhanced during the early stages of both acute exercise and its recovery, which might be associated with the mitochondrial stress and increased ROS generation. Given the above findings, we concluded that the mitochondrial function of the myocardium is adaptively regulated to meet the challenge of stress during acute exercise and its recovery period; however, ROS generation and the inflammatory reaction were simultaneously triggered and enhanced via the mitochondrial stress response.

Autophagy, an evolutionary pathway for the catabolism of cellar components, is closely associated with the lysosomal degradation of damaged organelles such as mitochondria [32]. When the autophagic process of selective degradation occurs within the mitochondria, it is known as mitophagy and an important regulated pathway that plays a crucial role in maintaining cellular homeostasis in various tissues [33]. Increasing evidence suggests that dysfunctional mitophagy is responsible for the deteriorations of human bodies associated with aging and disease [34, 35]. Recent studies have demonstrated that dysregulated mitophagy is involved in the pathogenesis of diabetic cardiomyopathy [36]. The current knowledge indicates that many positive and negative modulators of mitophagy, such as Beclin1, Atg14L, UVRAG, LC3, and Bnip3, tightly regulate the progression of mitophagy. AMP-activated protein kinase (AMPK), an essential energy sensor, regulates the induction of mitophagy via the phosphorylation of Beclin1 [37], whereas LC3 includes Pre-LC3, LC3 I, and LC3 II and plays a key regulatory role in the initiation of mitophagy on the autophagosome membrane [38]. Bnip3, a key regulatory factor for mitophagy, is essential for the formation of homodimers of Bnip3 with LC3 [39, 40].

To investigate the status of mitophagy in the myocardium during acute exercise and its recovery, we examined the expressions of several mitochondrial autophagy-related proteins including Beclin1, LC3, and Bnip3. Our data showed that these mitophagy-related proteins were all significantly upregulated during the early stages of both acute exercise and recovery. Noticeably, the pattern of the changes in these proteins is nearly the same as those for NLRP3 and IL-1*β*. These results suggest that the induction of mitophagy in the myocardium might be involved in the regulation of inflammatory reactions during the mitochondrial stress response. It has been demonstrated that accumulation of mitochondrial ROS can activate mitophagy and that mitophagy serves as a means for clearance of overproduced ROS [41]. Moreover, the blockade of mitophagy can result in the overgeneration of ROS and induce the activation of NLRP3 inflammasome in turn, which suggests that induced mitophagy mitigates inflammatory response via the negative regulation of ROS generation and NLRP3 inflammasome activation [9]. Thus, we suggest that the increased mitophagy observed in this study was stimulated to minimize myocardial injury via suppressing ROS-induced inflammatory response during acute exercise. Other studies have also revealed that mitophagy can be induced to various harmful stresses and proper induction of autophagy can maintain muscle homeostasis during exercise, which appears to be an adaptive response to protect against injury [42–44]. Additionally, the decreased mitochondrial function in E-120 versus RC might be associated with overstimulation of mitophagy in this study.

## 5. Conclusion

**Figure 7:**
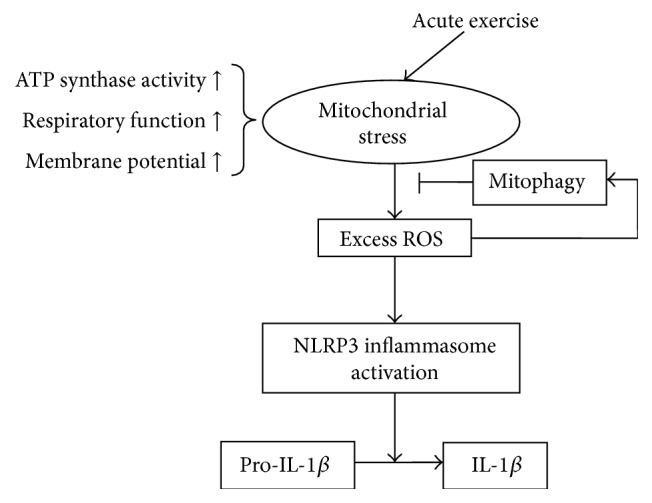
Acute exercise-induced mitochondrial stress triggers an inflammatory response in the myocardium via NLRP3 inflammasome activation with mitophagy.

In summary, we conclude that mitochondrial stress triggers the rat myocardium inflammatory response via activation of the NLRP3 inflammasome and induces mitophagy to minimize myocardial injury during acute exercise (Figure 7).
